# Supplementary material for: Explainable multi-task learning improves the parallel estimation of polygenic risk scores for many diseases through shared genetic basis
Source: PLoS Comput Biol. 2023 Jul 7;19(7):e1011211. doi: 10.1371/journal.pcbi.1011211 (PMC10328362; doi:10.1371/journal.pcbi.1011211)
Supplement: S1 Table — (PDF) [file pcbi.1011211.s001.pdf]

S1 Table: Comparison of STL and pan-disease MTL by ROC AUC and PR AUC for 60 non-cancer diseases with >0.5% prevalence.

Disease	Receiver operating characteristics (ROC) AUC#			Precision-recall (PR) AUC#			
	STL	Pan-disease MTL ROC		STL	Pan-disease MTL PR		Prevalence (Baseline)
		ROC AUC	Relative increase*		PR AUC	Relative increase*	
Allergy or anaphylactic reaction to food	54.35%	**55.52%**	**+26.80%**	0.61%	**0.64%**	**+44.67%**	0.54%
Angina	64.08%	**65.33%**	**+8.88%**	5.52%	**5.60%**	**+3.59%**	3.23%
Appendicitis	51.16%	**53.10%**	**+167.60%**	1.44%	**1.47%**	**+29.03%**	1.32%
Arthritis (nos)	55.17%	**55.64%**	**+9.02%**	1.51%	**1.52%**	**+6.25%**	1.22%
Asthma	60.01%	**61.15%**	**+11.39%**	16.44%	**17.02%**	**+12.37%**	11.75%
Atrial fibrillation	63.69%	**64.62%**	**+6.78%**	4.12%	**4.42%**	**+18.82%**	2.50%
Atrial flutter	64.05%	**64.69%**	**+4.59%**	3.60%	**3.70%**	**+6.62%**	2.12%
Back pain	52.75%	**53.44%**	**+24.94%**	1.15%	**1.19%**	**+34.87%**	1.03%
Benign breast lump	53.39%	**54.18%**	**+23.45%**	1.58%	**1.62%**	**+23.64%**	1.41%
Bronchitis	51.87%	**52.95%**	**+57.92%**	1.32%	**1.33%**	**+9.67%**	1.21%
Cataract	52.81%	**53.43%**	**+22.13%**	2.38%	**2.45%**	**+29.45%**	2.13%
Cervical spondylosis	56.00%	**58.97%**	**+49.47%**	0.87%	**1.02%**	**+95.82%**	0.72%
Chickenpox	53.73%	**54.16%**	**+11.41%**	2.58%	**2.62%**	**+20.03%**	2.33%
Gallstones	63.43%	**64.95%**	**+11.36%**	2.65%	**2.86%**	**+23.82%**	1.75%
Chronic sinusitis	53.23%	**53.92%**	**+21.31%**	0.82%	**1.07%**	**+343.92%**	0.75%
Depression	57.35%	**58.46%**	**+15.15%**	7.93%	**8.29%**	**+20.87%**	6.24%
Diabetes	67.26%	**67.92%**	**+3.83%**	9.75%	**9.99%**	**+4.78%**	4.59%
Dyspepsia / indigestion	53.67%	**55.05%**	**+37.66%**	3.13%	**3.34%**	**+64.05%**	2.80%
Ear/vestibular disorder	51.57%	**53.16%**	**+101.20%**	**1.01%**	1.00%	-7.92%	0.92%
Eczema/dermatitis	54.62%	**56.98%**	**+51.32%**	3.54%	**3.93%**	**+80.88%**	3.07%
Emphysema/chronic bronchitis	58.26%	**60.08%**	**+22.08%**	1.94%	**2.03%**	**+14.61%**	1.35%
Endometriosis	**54.13%**	53.80%	-7.88%	**1.77%**	1.75%	-9.67%	1.58%
Epilepsy	49.81%	**53.94%**	**+2188.46%**	0.81%	**0.93%**	**+1638.72%**	0.82%
Essential hypertension	51.92%	**54.37%**	**+127.01%**	1.38%	**1.47%**	**+78.20%**	1.26%
Eye/eyelid problem	49.52%	**50.96%**	**+298.50%**	0.79%	**0.84%**	**+711.13%**	0.80%
Fracture lower leg / ankle	53.99%	**54.65%**	**+16.63%**	0.56%	**0.60%**	**+89.87%**	0.52%
Gastro-oesophageal reflux (gord) / gastric reflux	53.91%	**55.18%**	**+32.31%**	5.57%	**5.73%**	**+22.40%**	4.87%
Glaucoma	58.48%	**59.31%**	**+9.86%**	1.65%	**1.83%**	**+42.36%**	1.24%
Gout	61.60%	**63.60%**	**+17.21%**	4.66%	**5.32%**	**+43.41%**	3.16%
Hayfever/allergic rhinitis	55.02%	**57.16%**	**+42.54%**	7.62%	**8.13%**	**+44.51%**	6.48%
Heart attack (MI)	71.25%	**72.56%**	**+6.16%**	5.06%	**5.27%**	**+7.30%**	2.30%
Hiatus hernia	55.90%	**57.71%**	**+30.64%**	3.06%	**3.26%**	**+36.16%**	2.51%
Hypertension	**62.89%**	61.78%	-8.64%	**37.70%**	36.41%	-12.40%	27.32%
Hyperthyroidism/thyrotoxicosis	67.77%	**71.55%**	**+21.27%**	1.74%	**2.14%**	**+45.39%**	0.85%
Hypothyroidism/myxoedema	73.90%	**74.96%**	**+4.43%**	12.99%	**13.59%**	**+7.53%**	5.14%
Inguinal hernia	75.09%	**76.31%**	**+4.89%**	1.49%	**1.57%**	**+9.41%**	0.66%
Iron deficiency anaemia	67.23%	**67.34%**	**+0.65%**	1.53%	**1.61%**	**+10.74%**	0.75%
Irritable bowel syndrome	62.16%	**62.38%**	**+1.78%**	**3.85%**	3.81%	-2.77%	2.56%
Joint pain	50.89%	**51.78%**	**+100.28%**	0.61%	**0.69%**	**+468.36%**	0.59%
Kidney stone/ureter stone/bladder stone	60.69%	**65.07%**	**+41.01%**	1.29%	**1.53%**	**+52.22%**	0.84%
Measles / morbillivirus	**54.81%**	53.73%	-22.39%	**2.06%**	1.97%	-34.87%	1.78%
Migraine	64.51%	**64.65%**	**+0.99%**	5.48%	**5.57%**	**+3.91%**	3.38%
Mumps / epidemic parotitis	55.02%	**55.73%**	**+14.19%**	1.23%	**1.33%**	**+85.88%**	1.11%
Oesophagitis/barretts oesophagus	54.94%	**55.57%**	**+12.66%**	2.57%	**2.60%**	**+6.79%**	2.16%
Osteoporosis	71.77%	**71.94%**	**+0.77%**	**3.67%**	3.65%	-1.38%	1.76%
Other joint disorder	50.51%	**50.55%**	**+8.05%**	**0.62%**	0.61%	-27.80%	0.60%
Pleural effusion	55.53%	**55.92%**	**+7.08%**	**0.78%**	0.77%	-2.67%	0.61%
Pneumonia	52.46%	**53.75%**	**+52.23%**	1.89%	**1.98%**	**+71.05%**	1.76%
Psoriasis	65.93%	**69.96%**	**+25.32%**	2.73%	**3.60%**	**+59.80%**	1.26%
PE +/- DVT	53.52%	**54.39%**	**+24.61%**	**1.12%**	1.04%	-33.39%	0.88%
Respiratory infection	53.23%	**55.10%**	**+58.13%**	2.15%	**2.21%**	**+22.05%**	1.88%
Rheumatoid arthritis	60.03%	**62.85%**	**+28.14%**	1.79%	**2.21%**	**+77.95%**	1.25%
Rubella / german measles	53.95%	**54.71%**	**+19.12%**	0.74%	**0.82%**	**+106.25%**	0.68%
Sciatica	52.14%	**53.54%**	**+65.85%**	1.25%	**1.33%**	**+65.47%**	1.14%
Stroke	59.06%	**59.80%**	**+8.11%**	2.14%	**2.27%**	**+23.21%**	1.58%
Tuberculosis (tb)	51.42%	**53.69%**	**+160.91%**	0.57%	**0.68%**	**+307.11%**	0.54%
Ulcerative colitis	57.43%	**60.72%**	**+44.32%**	0.77%	**0.77%**	**+4.13%**	0.55%
Urinary tract infection/kidney infection	**58.20%**	58.19%	-0.17%	**0.76%**	0.75%	-5.71%	0.58%
Varicose veins	58.09%	**59.84%**	**+21.72%**	0.71%	**0.76%**	**+27.42%**	0.52%
Whooping cough / pertussis	**52.32%**	51.15%	-50.68%	0.83%	**0.84%**	**+12.82%**	0.79%

#Best AUC highlighted in bold

*Relative increase = $\frac{(MTL\ AUC - baselin\ \ AUC)-(STL\ AUC - baseline\ AUC)}{STL\ AUC - baseline\ AUC} \times 100\%$
