# Supplementary material for: Explainable multi-task learning improves the parallel estimation of polygenic risk scores for many diseases through shared genetic basis
Source: PLoS Comput Biol. 2023 Jul 7;19(7):e1011211. doi: 10.1371/journal.pcbi.1011211 (PMC10328362; doi:10.1371/journal.pcbi.1011211)
Supplement: S2 Table — (PDF) [file pcbi.1011211.s002.pdf]

S2 Table: Numbers of shared important SNPs at 0.1% FDR between prevalent cancers in pan-cancer MTL.

Malignant melanoma	Non-melanoma skin cancer	Skin cancer	Lung cancer	Intrathoracic cancer	Colorectal cancer	Colon cancer	Rectal cancer	Bladder cancer	Uterine cancer	Cervical cancer	Prostate cancer	Breast cancer	Female genital tract cancer	Male genital tract cancer	Lymphoma	Non-hodgkins lymphoma	
	32	35	1	1	10	7	13	2	6	0	6	12	4	2	0	1	**Malignant melanoma**
		102	3	2	18	9	16	4	11	2	7	17	7	2	0	2	**Non-melanoma skin cancer**
			3	2	18	9	16	3	10	1	8	13	6	2	0	1	**Skin cancer**
				3	1	1	2	0	1	1	1	1	0	0	0	1	**Lung cancer**
					1	1	2	0	1	1	1	1	0	0	0	1	**Intrathoracic cancer**
						16	18	3	8	1	7	5	4	3	0	2	**Colorectal cancer**
							6	1	6	1	5	1	3	1	0	3	**Colon cancer**
								4	8	1	5	7	4	2	0	2	**Rectal cancer**
									2	0	1	0	0	0	0	0	**Bladder cancer**
										1	3	4	5	2	0	2	**Uterine cancer**
											1	0	3	1	0	1	**Cervical cancer**
												5	2	3	0	0	**Prostate cancer**
													2	1	0	0	**Breast cancer**
														2	0	1	**Female genital tract cancer**
															0	0	**Male genital tract cancer**
																0	**Lymphoma**
																	**Non-hodgkins lymphoma**
