# Supplementary material for: Explainable multi-task learning improves the parallel estimation of polygenic risk scores for many diseases through shared genetic basis
Source: PLoS Comput Biol. 2023 Jul 7;19(7):e1011211. doi: 10.1371/journal.pcbi.1011211 (PMC10328362; doi:10.1371/journal.pcbi.1011211)
Supplement: S1 Proof — (PDF) [file pcbi.1011211.s003.pdf]

S1 Proof

MTL LINA computes a $d \times 1$ output vector, Y, that contains the predicted states of d traits from an $m \times 1$ input vector, X, that contains the genotypes of m SNPs. MTL LINA can be expressed as:

$$Y = S(\boldsymbol{K} \cdot (A \circ X) + B),$$
$$A = F(X),$$

where $S(\,)$ is an activation function to be applied element-wise to its input column vector, $\boldsymbol{K}$ is a $d \times m$ coefficient matrix, A is a $m \times 1$ attention vector, B is a $d \times 1$ bias vector, $\cdot$ represents the matrix-vector multiplication, and $\circ$ represents the element-wise multiplication. For the binary classification phenotypes where $S(\,)$ is the sigmoid function, we define the $d \times 1$ vector $Logit$ as:

$$Logit = \boldsymbol{K} \cdot (A \circ X) + B$$

For any phenotype p we have:

$$Logit_p = K_p^T(A \circ X) + b_p$$

where K_p is the coefficient vector specific to phenotype p and b_p is the bias specific to phenotype p.

MTL LINA provides the first-order interpretation for phenotype p. For each phenotype, the gradient of the output logit, $Logit_p$, w.r.t the input feature X, is defined as the first-order importance score. The logit is used for the importance score computation because it produces more accurate results [1]. For phenotype p, the output gradient for feature $x_i \in X$ can be decomposed as follows:

$$\frac{\partial Logit_p}{\partial x_i} = k_{p,i}a_i + \sum_{j=1}^{m} k_{p,j}\frac{\partial a_j}{\partial x_i}x_j$$

where $k_{p,i} \in \boldsymbol{K}$, $x_j \in X$, and $(a_i, a_j) \in A^2$.

$\frac{\partial Logit_p}{\partial x_i}$ is the instance-wise first-order importance score of feature x_i for phenotype p.

The model-wise first-order importance score is derived as such:

$$\mathrm{FP_{p,i}} = \overline{\left|\frac{\partial Logit_p}{\partial x_i}\right|}$$

Where | | represents the absolute value operator and $\overline{}$ represents the mean operator.

MTL LINA provides the second-order interpretation for phenotype p. It is based on the second-order derivative for an attention neural network using the ReLU/Leaky-ReLU activation function in the hidden layers and the linear activation function in the attention layer. The second-order importance score between feature x_i and feature x_j for phenotype p is expressed as:

$$\frac{\partial^2 Logit_p}{\partial x_i \partial x_j} = k_{p,j}\frac{\partial a_j}{\partial x_i} + k_{p,i}\frac{\partial a_i}{\partial x_j}$$

where $\left(k_{p,i}, k_{p,j}\right) \in \boldsymbol{K}^2$, $\left(x_i, x_j\right) \in X^2$, and $\left(a_i, a_j\right) \in A^2$.

The instance-wise second-order importance score for a feature pair (x_i, x_j) w.r.t. a phenotype p is defined as their second-order derivative. The model-wise second-order importance score is defined as:

$$\mathrm{SP_{p,i,j}} = \overline{\left|\frac{\partial^2 Logit_p}{\partial x_i \partial x_j}\right|}$$

Here, we demonstrate that the second-order derivative, under the condition of ReLU/Leaky-ReLU as the activation function in the hidden layers, can be derived as:

$$\frac{\partial^2 Logit_p}{\partial x_i \partial x_j} = k_{p,j}\frac{\partial a_j}{\partial x_i} + k_{p,i}\frac{\partial a_i}{\partial x_j}$$

where $(k_{p,i}, k_{p,j}) \in \boldsymbol{K}^2$, $(x_i, x_j) \in X^2$, and $(a_i, a_j) \in A^2$.

Proof:

$$\frac{\partial^2 Logit_p}{\partial x_i \partial x_j} = K_p \begin{bmatrix} x_1 \frac{\partial^2 a_1}{\partial x_i \partial x_j} \\ \vdots \\ x_{i-1} \frac{\partial^2 a_{i-1}}{\partial x_i \partial x_j} \\ \boldsymbol{x_i \frac{\partial^2 a_i}{\partial x_i \partial x_j} + \frac{\partial a_i}{\partial x_j}} \\ x_{i+1} \frac{\partial^2 a_{i+1}}{\partial x_i \partial x_j} \\ \vdots \\ x_{j-1} \frac{\partial^2 a_{j-1}}{\partial x_i \partial x_j} \\ \boldsymbol{x_j \frac{\partial^2 a_j}{\partial x_i \partial x_j} + \frac{\partial a_j}{\partial x_i}} \\ x_{j+1} \frac{\partial^2 a_{j+1}}{\partial x_i \partial x_j} \\ \vdots \\ x_n \frac{\partial^2 a_n}{\partial x_i \partial x_j} \end{bmatrix}$$

where $K_p \in \boldsymbol{K}$ is the coefficient vector for phenotype p.

We aim to demonstrate that, for any neuron, q, in the attention layer that outputs A (*i.e.*, $q \in A$)

$$\frac{\partial^2 a_q}{\partial x_i \partial x_j} = 0 \text{ for any } x_i, x_j.$$

For any neuron $q \in A$:

$$a_q = \sum_{k=1}^{m_l} w_{q,k,l} f_{k,l}$$

$$\frac{\partial a_q}{\partial x_j} = \sum_{k=1}^{m_l} w_{q,k,l} \frac{\partial f_{k,l}}{\partial x_j}$$

$$\frac{\partial^2 a_q}{\partial x_i \partial x_j} = \sum_{k=1}^{m_l} w_{q,k,l} \frac{\partial^2 f_{k,l}}{\partial x_i \partial x_j}$$

where $f_{k,l}$ is the activation function output from neuron k on hidden layer l containing m_l neurons, and $w_{i,k,l}$ the coefficient of the connection between neuron q on layer A and neuron k on layer l.

For this proof, we define the activation functions:

ReLU$(x) = \begin{cases} x, & if\ x > 0 \\ 0, & else \end{cases}$

and Leaky-ReLU$(x) = \begin{cases} x, & if\ x > 0 \\ -\alpha x, & else \end{cases}$, where α is a constant.

Initial case:

Let's assume the case where MTL LINA has only one hidden layer.

For any neuron q on the 1st hidden layer, we have:

$$\frac{\partial f_{q,1}}{\partial x_j} = \frac{\partial f_{q,1}}{\partial o_{q,1}} \frac{\partial o_{q,1}}{\partial x_j}$$

With $o_{q,1}$ being the output of neuron q before activation.

$$o_{q,1} = \sum_{k=1}^{m} w_{q,k,1} x_k$$

Because $w_{q,k,1}$ is independent of x_j,

$$\frac{\partial o_{q,1}}{\partial x_j} = \sum_{k=1}^{m} w_{q,k,1} \frac{\partial x_k}{\partial x_j}$$

Then:

$$\frac{\partial f_{q,1}}{\partial x_j} = \frac{\partial f_{q,1}}{\partial o_{q,1}} \sum_{k=1}^{m} w_{q,k,1} \frac{\partial x_k}{\partial x_j}$$

$$\frac{\partial f_{q,1}}{\partial x_j} = \frac{\partial f_{q,1}}{\partial o_{q,1}} w_{q,j,1}$$

Then:

$$\frac{\partial^2 f_{q,1}}{\partial x_i \partial x_j} = \frac{\partial}{\partial x_i}\left(\frac{\partial f_{q,1}}{\partial o_{q,1}} w_{q,j,1}\right)$$

$$\frac{\partial^2 f_{q,1}}{\partial x_i \partial x_j} = w_{q,j,1} \frac{\partial}{\partial x_i}\left(\frac{\partial f_{q,1}}{\partial o_{q,1}}\right)$$

When $f_{q,1}$ is ReLU or leaky-ReLU, then $\frac{\partial}{\partial x_i}\left(\frac{\partial f_{q,1}}{\partial o_{1,1}}\right) = 0$ because for ReLU: $\frac{\partial f_{q,1}}{\partial o_{q,1}} = \begin{cases} 1, \ if \ f_{q,1} > 0 \\ 0, \ else \end{cases}$ or Leaky-ReLU: $\frac{\partial f_{q,1}}{\partial o_{q,1}} = \begin{cases} 1, \ if \ f_{q,1} > 0 \\ -\alpha, \ else \end{cases}$ and so the second-order derivative of those functions is assumed to be 0 everywhere. Thus:

$$\frac{\partial^2 f_{q,1}}{\partial x_i \partial x_j} = 0$$

And:

$$\frac{\partial^2 a_q}{\partial x_i \partial x_j} = \sum_{k=1}^{m_1} w_{q,k,1} \frac{\partial^2 f_{k,1}}{\partial x_i \partial x_j} = 0$$

Induction:

We hypothesize that, for a neural network with 2 or more hidden layers, we have at layer l, for any neuron q:

$$\frac{\partial^2 f_{q,l}}{\partial x_i \partial x_j} = 0$$

On the next hidden layer $l+1$, we have, for any neuron q:

$$\frac{\partial f_{q,l+1}}{\partial x_j} = \frac{\partial f_{q,l+1}}{\partial o_{q,l+1}} \frac{\partial o_{q,l+1}}{\partial x_j}$$

And:

$$o_{q,l+1} = \sum_{k=1}^{m_l} w_{q,k,l}\, f_{k,l}$$

Because $w_{q,k,l}$ is independent of x_j:

$$\frac{\partial o_{q,l+1}}{\partial x_j} = \sum_{k=1}^{m_l} w_{q,k,l} \frac{\partial f_{k,l}}{\partial x_j}$$

Then:

$$\frac{\partial f_{q,l+1}}{\partial x_j} = \frac{\partial f_{q,l+1}}{\partial o_{q,l+1}} \sum_{k=1}^{m_l} w_{q,k,l} \frac{\partial f_{k,l}}{\partial x_j}$$

$$\frac{\partial^2 f_{q,l+1}}{\partial x_i \partial x_j} = \frac{\partial}{\partial x_i}\left(\frac{\partial f_{q,l+1}}{\partial o_{q,l+1}} \sum_{k=1}^{m_l} w_{q,k,l} \frac{\partial f_{k,l}}{\partial x_j}\right)$$

$$\frac{\partial^2 f_{q,l+1}}{\partial x_i \partial x_j} = \frac{\partial f_{q,l+1}}{\partial o_{q,l+1}} \sum_{k=1}^{m_l} w_{q,k,l} \frac{\partial}{\partial x_i}\left(\frac{\partial f_{k,l}}{\partial x_j}\right) + \frac{\partial}{\partial x_i}\left(\frac{\partial f_{q,l+1}}{\partial o_{q,l+1}}\right) \sum_{k=1}^{m_l} w_{q,k,l} \frac{\partial f_{k,l}}{\partial x_j}$$

$\frac{\partial}{\partial x_i}\left(\frac{\partial f_{q,l+1}}{\partial o_{q,l+1}}\right) = 0$ because the second derivative of ReLU or Leaky-ReLU is zero.

Thus,

$$\frac{\partial^2 f_{q,l+1}}{\partial x_i \partial x_j} = \frac{\partial f_{q,l+1}}{\partial o_{q,l+1}} \sum_{k=1}^{m_l} w_{q,k,l} \frac{\partial}{\partial x_i}\left(\frac{\partial f_{k,l}}{\partial x_j}\right)$$

For any neuron q on l (hypothesis):

$$\frac{\partial^2 f_{q,l}}{\partial x_i \partial x_j} = 0$$

By deduction:

$$\frac{\partial^2 f_{q,l+1}}{\partial x_i \partial x_j} = \frac{\partial f_{q,l+1}}{\partial o_{q,l+1}} \sum_{k=1}^{m_l} w_{q,k,l}\, 0$$

$$\boldsymbol{\frac{\partial^2 f_{q,l+1}}{\partial x_i \partial x_j} = 0}$$

Conclusion:

By induction we have demonstrated that for any neuron q on any layer l:

$$\boldsymbol{\frac{\partial^2 f_{q,l}}{\partial x_i \partial x_j} = 0}$$

Therefore,

$$\frac{\partial^2 a_q}{\partial x_i \partial x_j} = \sum_{k=1}^{m_l} w_{q,k,l} \frac{\partial^2 f_{k,l}}{\partial x_i \partial x_j} = \sum_{k=1}^{m_l} w_{q,k,l} 0$$

$$\boldsymbol{\frac{\partial^2 a_q}{\partial x_i \partial x_j} = 0}$$

For any $a_q \in A$

$$\frac{\partial^2 Logit_p}{\partial x_i \partial x_j} = K_p^T \begin{bmatrix} 0 \\ \vdots \\ 0 \\ \boldsymbol{\frac{\partial a_i}{\partial x_j}} \\ 0 \\ \vdots \\ 0 \\ \boldsymbol{\frac{\partial a_j}{\partial x_i}} \\ 0 \\ \vdots \\ 0 \end{bmatrix}$$

Hence:

$$\frac{\partial^2 Logit_p}{\partial x_i \partial x_j} = k_{p,j} \frac{\partial a_j}{\partial x_i} + k_{p,i} \frac{\partial a_i}{\partial x_j}$$

<u>End-of-proof</u>

<u>Reference:</u>

[1] K. Simonyan, A. Vedaldi, and A. Zisserman, “Deep Inside Convolutional Networks: Visualising Image Classification Models and Saliency Maps.” arXiv, Apr. 19, 2014. doi: 10.48550/arXiv.1312.6034.
